# Reduced antiviral gene expression and elevated CXCL8 expression in peripheral blood are associated with severe hypoxemia in RSV-infected children

**DOI:** 10.3389/fimmu.2024.1438630

**Published:** 2024-09-30

**Authors:** Carlos Pita-Martínez, Carmen Goez-Sanz, Ana Virseda-Berdices, Alejandro Gonzalez-Praetorius, Esther Mazario-Martín, María Rodriguez-Mesa, Marta Quero-Delgado, Vanesa Matías, Isidoro Martínez, Salvador Resino

**Affiliations:** ^1^ Unidad de Infección Viral e Inmunidad, Centro Nacional de Microbiología, Instituto de Salud Carlos III, Majadahonda, Spain; ^2^ Gerencia de Atención Primaria Valladolid Oeste, Centro de Salud Delicias II, Valladolid, Spain; ^3^ Servicio de Pediatría, Hospital Clínico Universitario de Valladolid, Valladolid, Spain; ^4^ Centro de Investigación Biomédica en Red en Enfermedades Infecciosas (CIBERINFEC), Instituto de Salud Carlos III, Madrid, Spain; ^5^ Servicio de Microbiología, Hospital Universitario de Guadalajara, Guadalajara, Spain; ^6^ Servicio de Pediatría, Hospital Universitario de Guadalajara, Guadalajara, Spain; ^7^ Servicio de Pediatría, Hospital Universitario Infanta Cristina, Parla, Spain

**Keywords:** respiratory syncytial virus, children, bronchiolitis, gene expression, immune response, peripheral blood, hypoxemia

## Abstract

The pathology of respiratory syncytial virus (RSV) infection remains unclear. An unbalanced immune response to RSV infection can lead to immunopathology, causing airway damage and impaired exchange of oxygen and carbon dioxide between the air and the bloodstream. We aimed to evaluate the association of the expression of inflammatory and antiviral genes in peripheral blood with severe hypoxemia in children with RSV infection seen in the hospital emergency room. We conducted a cross-sectional study on 121 RSV-infected children seen in hospital emergency rooms between 2015 and 2023. Total RNA was extracted from whole blood samples, and gene expression (*IL-6*, *TNFα*, *CXCL8*, *ISG15*, *IFIT1*, *RIGI*, *IFNβ*, *CCL5*, and *CXCL10*) was quantified using quantitative RT-PCR. The outcome variable was having severe hypoxemia (SpO_2_ ≤ 90%). The association analysis was performed using a volcano plot, adjusted logistic regression, and orthogonal partial least squares discriminant analysis (OPLS-DA). We found that 26 of 121 children had severe hypoxemia (SpO_2_ ≤ 90%). *CXCL8* was overexpressed [fold changes (FC) > 2; *q*-value < 0.05], and *ISG15*, *IFIT1*, *RIGI*, *IFNβ*, *CCL5*, and *CXCL10* were underexpressed (FC <0.5; *q*-value <0.05) in children with severe hypoxemia. These associations were ratified using adjusted logistic regression. The OPLS-DA showed that the gene expressions of *CXCL8*, *ISG15*, *IFIT1*, *RIGI*, and *CXCL10* had values of variable importance in projection (VIP) ≥1, being the most relevant features. In conclusion, an imbalance favoring inflammation over antiviral defense may contribute to the pathogenesis of severe hypoxemia in RSV-infected children. These findings provide valuable insights into the pathology of RSV infection.

## Graphical Abstract

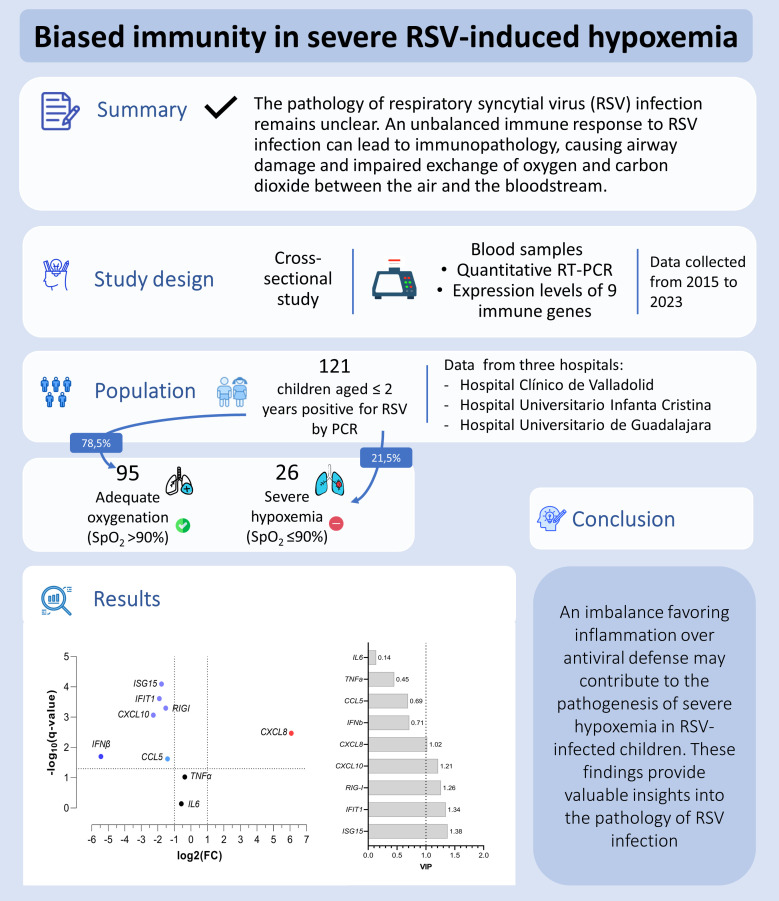

## Introduction

Respiratory syncytial virus (RSV) is a leading cause of acute lower respiratory tract infection (LRTI) in children, resulting in a major global health burden (1). Clinical manifestations range from asymptomatic or mild upper respiratory infections to severe LRTI, requiring intensive care (2). Saturation of peripheral oxygen (SpO_2_) in the emergency department is an effective method for the initial evaluation of RSV bronchiolitis, as it closely relates to cyanosis and disease severity, reflected in maximum oxygen requirements. An SpO_2_ ≤90% is considered severe hypoxemia, which is significantly associated with severe bronchiolitis and increased risk of death (3).

Antiviral strategies for RSV focus on prophylaxis and treatment of severe cases. Ribavirin, a nebulized antiviral for high-risk infants and immunocompromised patients with severe RSV lower respiratory tract infections, has debated efficacy and limitations due to cost and side effects. Palivizumab, a preventive monoclonal antibody for high-risk infants, reduces severe RSV disease but warrants cost-effectiveness evaluation (4). Recently, a vaccine based on the RSV prefusion F protein (ABRYSVO) has been approved for pregnant women (5). Also, an improved anti-F monoclonal antibody (Beyfortus) is now available to prevent RSV bronchiolitis in neonates and infants during their first RSV season (6). Vaccinating mothers with ABRYSVO and providing neonatal prophylaxis with Beyfortus aim to reduce RSV severity in newborns by providing protective antibodies. However, ABRYSVO or Beyfortus does not always offer complete protection, and a child can still contract RSV. Furthermore, combining maternal vaccination and neonatal antibody administration could be fine-tuned using these markers to optimize protection and minimize adverse effects.

The role of the immune response in the etiology of RSV bronchiolitis is increasingly recognized, as it plays a critical role in effective viral control and clearance (7). RSV replication generates double-stranded RNA (dsRNA) and single-stranded RNA (ssRNA) carrying an uncapped 5′ triphosphate, which act as pathogen-associated molecular patterns (PAMPs), stimulating toll-like receptors (TLRs) and retinoic acid-inducible gene I (RIGI) (7). This recognition triggers intracellular signaling pathways that lead to the activation of multiple transcription factors, such as interferon regulatory factors 3 and 7 (IRFs), activating protein (AP-1), and nuclear factor-κB (NF-κB) (7, 8). Subsequent translocation of these factors to the nucleus induces the expression of several proinflammatory cytokines, chemokines, and interferons (IFN) (types I and III), which are involved in the antiviral response and inflammation (7, 8). IFN-mediated signaling, in turn, upregulates the expression of numerous IFN-stimulated genes (ISGs) and establishes an antiviral state with the aim of restricting viral replication. This initial innate immune response is also essential in triggering an effective adaptive antiviral systemic immunity.

However, the mechanisms contributing to the pathology of RSV infection remain unclear, and an unbalanced immune response against RSV infection may lead to immunopathology, resulting in airway damage (7). In contrast, a robust innate immune response that effectively controls virus spreading appears to be associated with milder RSV disease (7). Ethnicity also influences the immune response to RSV, affecting infection rates, severity, and outcomes (9). These differences are mainly due to genetic, environmental, socioeconomic, and cultural factors.

Studying pulmonary immunity against RSV infection is complicated by its difficult access. However, peripheral blood can serve as a liquid biopsy due to the close connection between the circulatory and respiratory systems (10, 11). Liquid biopsy allows a wide range of cellular and molecular assays that evaluate the underlying organ through minimally invasive techniques (12). These assays evaluate biomarkers useful for diagnosis, prognosis, and treatment of diseases (13, 14).

### Objective

We aimed to evaluate the association of the expression of inflammatory and antiviral genes in peripheral blood with severe hypoxemia in children with RSV infection seen in the hospital emergency room.

## Methods

### Study design and patients

We conducted a cross-sectional study on 121 children under 2 years of age infected with RSV between 2015 and 2023 in three Spanish hospitals: Hospital Clínico Universitario de Valladolid, Hospital Universitario Infanta Cristina, and Hospital Universitario de Guadalajara, Spain. All children presented to the hospital emergency room with acute RSV infection.

The research was done according to the Declaration of Helsinki. All infants’ parents or legal guardians provided informed consent. It was approved by the Ethics Committee of Instituto de Salud Carlos III (PI 84_2015-v2) and the Institutional Review Board of the respective hospitals (November 22, 2018; Title: Control of the balance between inflammation and viral replication in human respiratory syncytial virus infections).

### Children’s data and outcome variable

Epidemiological, clinical, and analytical data were collected from medical records. Babies born before the 37th week of gestation were considered premature.

All children tested positive for RSV. Combined nasopharyngeal and oropharyngeal swabs were collected in the emergency room, and these biological samples were sent to the Microbiology Laboratory of each hospital for the diagnosis of respiratory infection by polymerase chain reaction (PCR) testing, according to the protocols previously implemented in each laboratory for hospital care.

Patients were classified according to the severity of RSV bronchiolitis as mild, moderate, and severe, according to the Wood-Downes score (WDS) (15) and the Bronchiolitis de Sant Joan de Déu score (BROSJDD) (16) (**Supplementary Tables 1**, **2**).

The outcome variable was severe hypoxemia (SpO_2_ ≤ 90%), evaluated by pulse oximetry during initial vital signs recorded upon arrival at the emergency room. SpO_2_ for each participant was the first value collected.

### Quantitative RT-PCR assay for blood biomarkers

Blood samples were collected in tubes containing EDTA within the first 24 h of admission to the emergency department and stored at −80°C.

NucleoSpin RNA Kit (Macherey-Nagel, Düren, Germany) was used to extract total RNA and the High-Capacity cDNA Reverse Transcription Kit (Applied Biosystems, Foster City, CA, USA) to transcribe to cDNA. The selected genes were *Actin-β* (ACTB; Hs99999903_m1), *interleukin 6* (IL6; Hs00985639_m1), *tumor necrosis factor-alpha* (TNFα; Hs00174128_m1), *chemokine C-X-C motif ligand 8* (IL8/CXCL8; Hs00174103_m1), *interferon-stimulated gene 15* (ISG15; Hs00192713_m1), *interferon-induced protein with tetratricopeptide repeats 1* (*IFIT1*; Hs03027069_s1), *retinoic acid-inducible gene I* (RIGI; Hs00204833_m1), *interferon-β1* (IFNB1; Hs01077958_s1), *chemokine C-C motif ligand 5* (CCL5; Hs00982282_m1), and *chemokine C-X-C motif ligand 10* (CXCL10; Hs00171042_m1). The gene expression was measured by real-time PCR (RT-PCR) using the TaqMan Gene Expression Assays (Applied Biosystems, Foster City, CA, USA). PCR assays were performed in triplicate using 48-well plates and a StepOne RT-PCR System thermal cycler (Applied Biosystems). Differential expression analysis was performed by the Ct (cycle threshold) (ΔΔCT) method, using *ACTB* as endogenous control. A reference sample of total RNA extracted from RSV-infected A549 cells was used as the calibrator. Gene expression levels were determined relative to the calibrator.

### Statistical analysis

Clinical and epidemiological characteristics were compared using the Mann–Whitney *U* test for continuous variables and the Pearson chi-square test (*χ* ^2^) or Fisher’s exact test for categorical variables.

Peripheral blood gene expression values were normalized (*Z*-score) using log transformation (log_10_) and scaling by mean centering (**Supplementary Figure 1**). Next, normalized gene expression was compared between groups using a *t*-test and a volcano plot that integrated fold changes (FCs) and *t*-test *q*-values. The association between normalized gene expression values and the outcome variable was evaluated using logistic regressions, adjusted by the most relevant clinical covariables (age, gender, prematurity, RSV bronchiolitis severity, and days since onset of respiratory symptoms). These covariables were selected through a stepwise algorithm, with input and output *p*-values of 0.15 and 0.20, respectively. This approach provided adjusted odds ratio (aOR) and 95% confidence intervals (CIs). Finally, we performed a supervised multivariate analysis of all gene expression data using orthogonal partial least squares discriminant analysis (OPLS-DA). The model was validated using a permutation test (*n* = 1,000), resulting in R2Y and Q2 values as performance indicators. The OPLS-DA also provided the variable importance in projection (VIP) score for each feature, with VIP ≥1 indicating significant variables.

Statistical analysis was performed using Stata IC 17 (StataCorp, TX, USA) and MetaboAnalyst 6.0 software (http://www.metaboanalyst.ca/). GraphPad Prism 9.0 (GraphPad Software, Inc., San Diego, CA, USA) was used to make the graphs. All *p*-values were two-tailed, the significance level was set at 0.05, and raw *p*-values were adjusted for multiple testing using the false discovery rate (FDR, *q*-value).

## Results

### Patient characteristics

**Table 1** shows the clinical characteristics of RSV-infected children in the emergency room, where 55.4% of them were male children, the median age was 8.7 weeks, and 40.8% were less than 6 weeks old. The median weight percentile was 45, and the height percentile was 57. The median gestation age was 39 weeks, and prematurity (<37 weeks) was 13.4%. The onset of respiratory symptoms was 3 days before admission, and bronchiolitis was 33.9% mild, 62.8% moderate, and 3.3% severe. We only had ethnic data on 72.7% (88/121) of the patients. Of these, 57 were of Caucasian origin, 12 were Gypsies, 9 were Latin American, 8 were Maghreb, one was sub-Saharan African, and one was Asian.

**Table 1:** Summary of characteristics of RSV-infected children according to oxygen saturation (SpO_2_) levels above or below 90%.

Characteristic	All	Non-severe hypoxemia (SpO_2_ > 90%)	Severe hypoxemia (SpO_2_ ≤ 90%)	*p*-value
**No. of patients**	121	95	26	
**Age (weeks), median (IQR)**	8.7 (4.4–17.4.3)	8.7 (4.4–21.8)	6.6 (4.3–13.1)	0.475
<6 weeks, *N* (%)	49 (40.8%)	38 (40.0%)	11 (44%)	0.717
**Gender (male), *N* (%)**	67 (55.4%)	56 (52.6%)	11 (14.4%)	0.130
**Weight percentile, median (IQR)**	45 (26–73)	46 (28–71)	33.5 (19–74.5)	0.582
**Height percentile, median (IQR)**	57 (33–78)	62 (38–79)	40 (26.5–68)	0.125
**Breastfeeding, N (%)**	91 (79.8%)	73 (82.0%)	18 (72.0%)	0.270
**Gestation age (weeks), median (IQR)**	39 (38–40)	39 (38–40)	40 (38–40)	0.686
Prematurity, *N* (%)				
<37 weeks	15 (13.4%)	12 (13.5%)	3 (13%)	0.999
≤34 weeks	4 (3.3%)	3 (3.2%)	1 (3.8%)	0.999
**Respiratory symptoms onset (days), median (IQR)**	3 (2–4)	3 (2–4)	2 (2–4)	0.208
Bronchiolitis severity, *N* (%)				
Mild	41 (33.9%)	30 (31.6%)	11 (42.2%)	0.306
Moderate	76 (62.8%)	64 (67.4%)	12 (46.2%)	**0.047**
Severe	4 (3.3%)	1 (1%)	3 (11.6%)	**0.008**

Statistics: Values are expressed as the median (interquartile range) for continuous variables and absolute count (percentage) for categorical variables. The statistically significant differences are shown in bold.

IQR, interquartile range; RSV, Respiratory syncytial virus.

Moreover, 26 of 121 children had severe hypoxemia (SpO_2_ ≤ 90%). Significant differences in bronchiolitis severity were found between children according to their SpO_2_ levels (above or below 90%).

### Peripheral blood gene expression and severe hypoxemia

**Figure 1:**
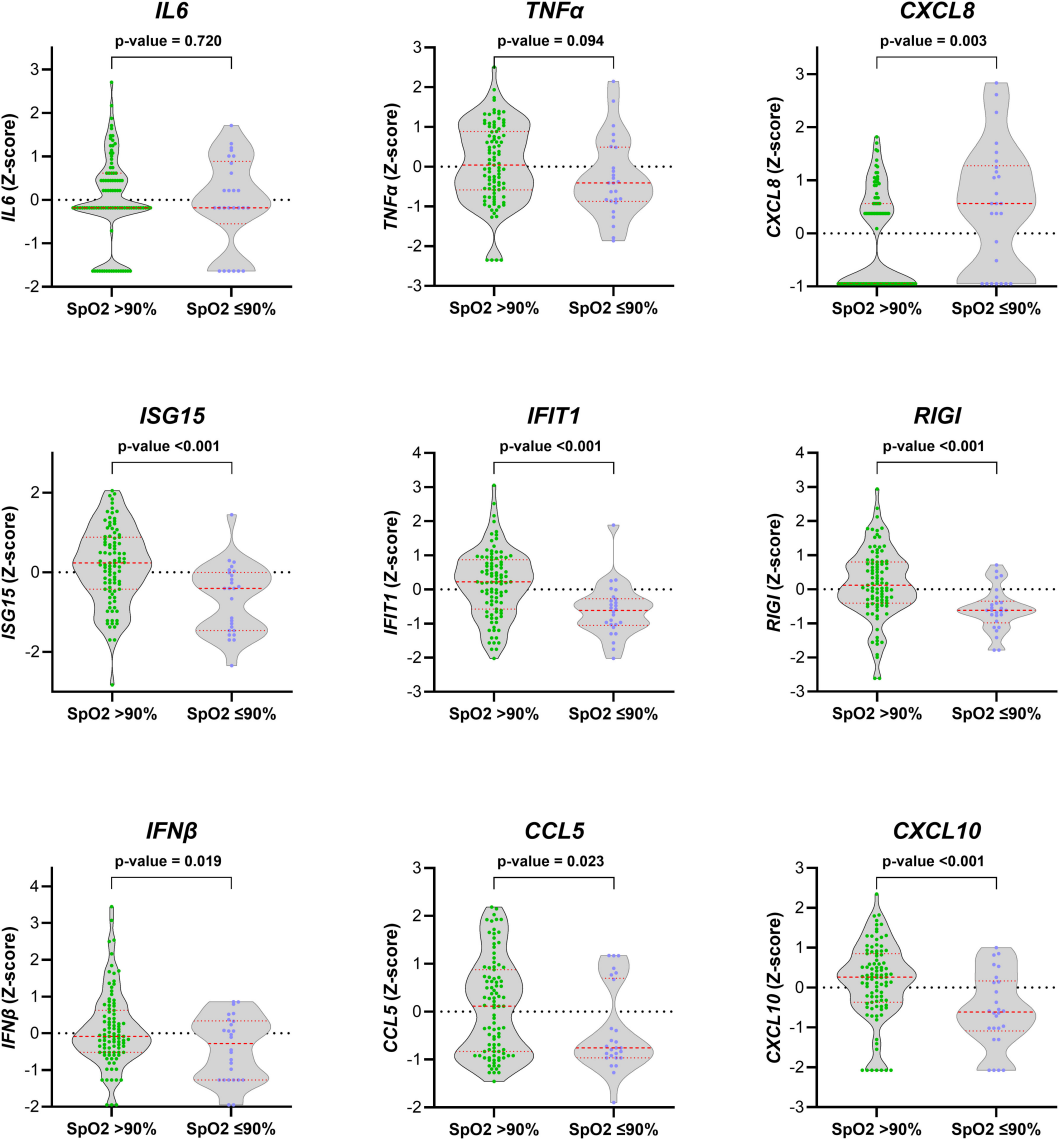
Normalized values (*Z*-score) of peripheral blood gene expression according to the severity of hypoxemia (SpO_2_ > 90% vs. SpO_2_ ≤ 90%) in RSV-infected children. Statistics: Gene expression values were normalized using log transformation (log_10_) and scaling by mean centering. Differences between groups were assessed using a *t*-test. RSV, respiratory syncytial virus; SpO_2_, saturation of peripheral oxygen; *p*-value, raw significance level; IL6, interleukin 6; TNFα, tumor necrosis factor-alpha; CXCL8, chemokine C-X-C motif ligand 8; ISG15, interferon-stimulated gene 15; IFIT1, interferon-induced protein with tetratricopeptide repeats 1; RIGI, retinoic acid-inducible gene I; IFNβ, interferon-β1; CCL5, chemokine C-C motif ligand 5; CXCL10, chemokine C-X-C motif ligand 10.

**Figure 2:**
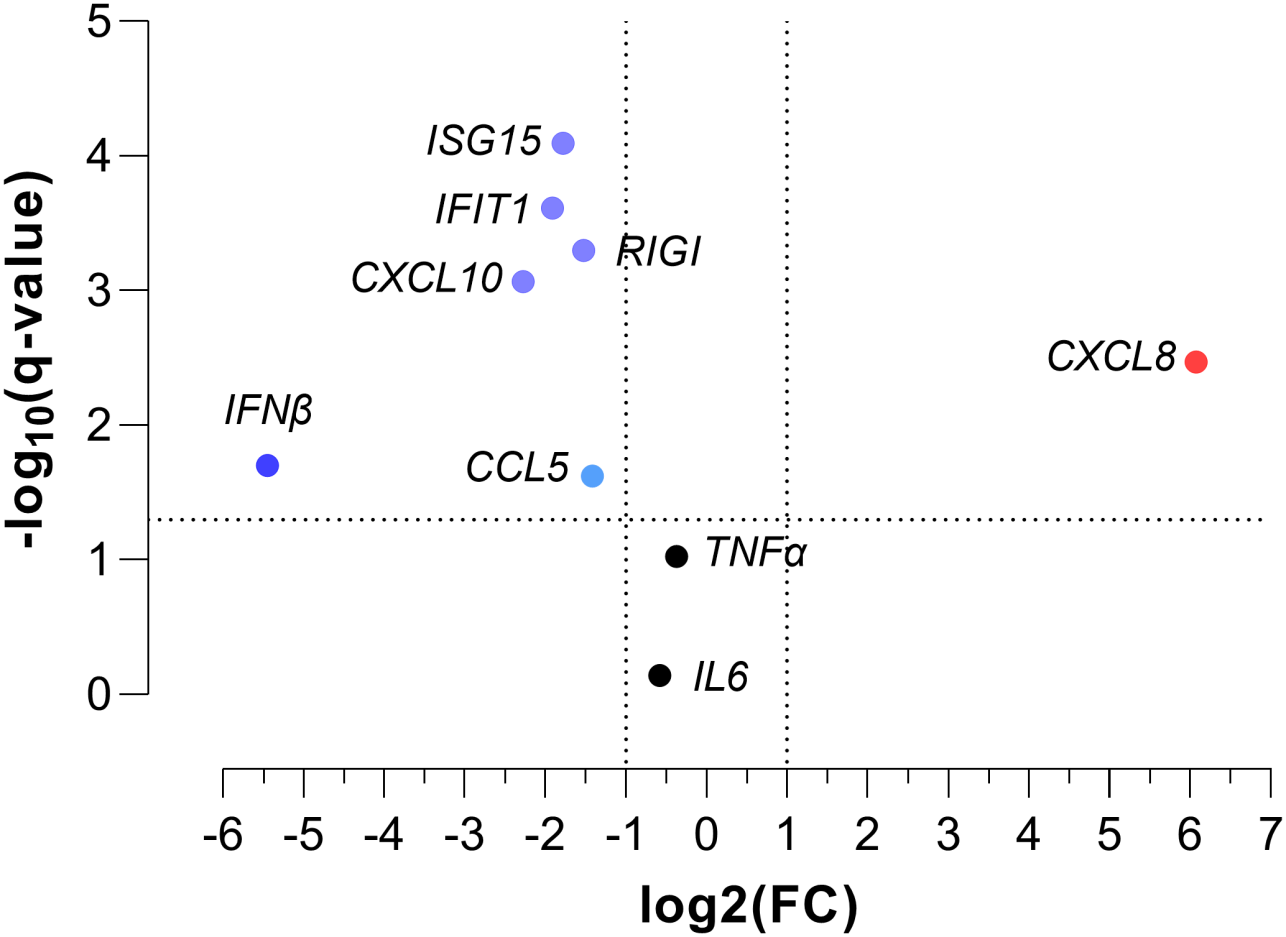
Volcano plot of normalized peripheral blood gene expression values for severe hypoxemia (SpO_2_ ≤ 90%) in RSV-infected children. Statistics: Gene expression values were normalized using log transformation (log_10_) and scaling by mean centering. Differences between groups were assessed using a *t*-test, and *p*-values were corrected for multiple tests using the false discovery rate (FDR, *q*-values). RSV, respiratory syncytial virus; SpO_2_, saturation of peripheral oxygen; FC, fold change; *q*-value, adjusted significance level; IL6, interleukin 6; TNFα, tumor necrosis factor-alpha; CXCL8, chemokine C-X-C motif ligand 8; ISG15, interferon-stimulated gene 15; IFIT1, interferon-induced protein with tetratricopeptide repeats 1; RIGI, retinoic acid-inducible gene I; IFNβ, interferon-β1; CCL5, chemokine C-C motif ligand 5; CXCL10, chemokine C-X-C motif ligand 10.

**Figure 3:**
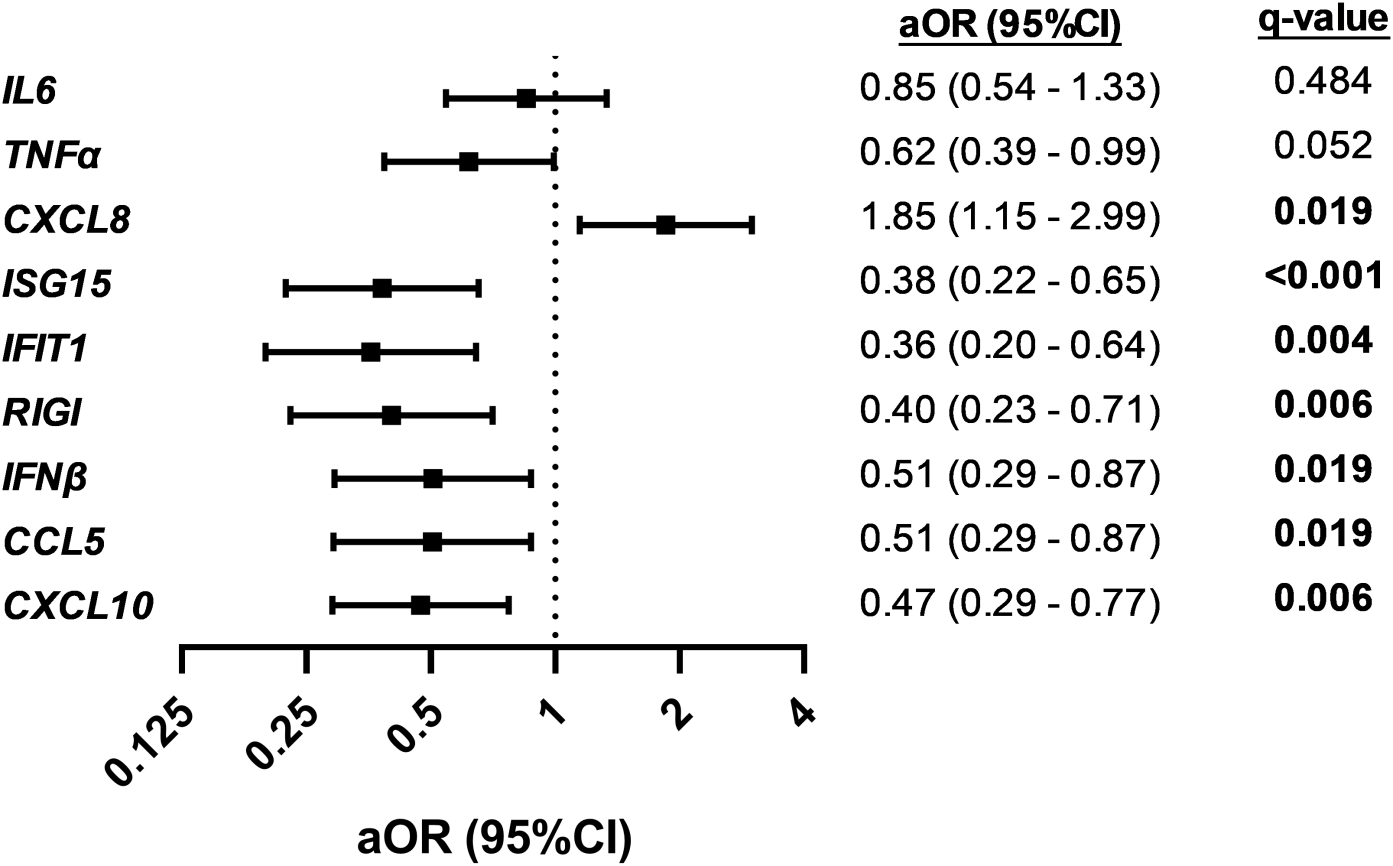
Association between normalized peripheral blood gene expression values and severe hypoxemia (SpO_2_ ≤ 90%) in RSV-infected children. Statistics: Data were calculated using logistic regression adjusted by clinical covariables (age, gender, prematurity, RSV bronchiolitis severity, and days since onset of respiratory symptoms). The *p*-values were corrected for multiple testing using the false discovery rate (FDR, *q*-values). Significant differences are shown in bold. RSV, respiratory syncytial virus; SpO_2_, saturation of peripheral oxygen; aOR, adjusted odds ratio; 95% CI, 95% of confidence interval; *q*-value, corrected significance level; IL6, interleukin 6; TNFα, tumor necrosis factor-alpha; CXCL8, chemokine C-X-C motif ligand 8; ISG15, interferon-stimulated gene 15; IFIT1, interferon-induced protein with tetratricopeptide repeats 1; RIGI, retinoic acid-inducible gene I; IFNβ, interferon-β1; CCL5, chemokine C-C motif ligand 5; CXCL10, chemokine C-X-C motif ligand 10.

We found significant differences in the gene expression of *CXCL8*, *ISG15*, *IFIT1*, *RIGI*, *IFNβ*, *CCL5*, and *CXCL10* between children with SpO_2_ >90% and children with SpO_2_ ≤90% (*p*-value < 0.05; **Figure 1**). *CXCL8* was overexpressed (FC > 2; *q*-value < 0.05), and *ISG15*, *IFIT1*, *RIGI*, *IFNβ*, *CCL5*, and *CXCL10* were underexpressed (FC <0.5; *q*-value <0.05) in children with severe hypoxemia (**Figure 2**). These results were ratified using logistic regression adjusted by clinical characteristics and FDR (**Figure 3**). High values of *CXCL8* (aOR = 1.85; *q*-value = 0.019) were directly associated with severe hypoxemia, while *ISG15* (aOR = 0.38; *q*-value < 0.001), *IFIT1* (aOR = 0.36; *q*-value = 0.004), *RIGI* (aOR = 0.40; *q*-value = 0.006), *IFNβ* (aOR = 0.51; *q*-value = 0.019), *CCL5* (aOR = 0.51; *q*-value = 0.019), and *CXCL10* (aOR = 0.47; *q*-value = 0.006) were inversely associated with severe hypoxemia (**Figure 3**).

Finally, we performed a supervised multivariate dimensionality reduction for all peripheral blood gene expression with an OPLS-DA validated by a permutation test (*p* < 0.05; **Supplementary Figure 2**). The OPLS-DA showed that the gene expressions of *CXCL8*, *ISG15*, *IFIT1*, *RIGI*, and *CXCL10* had values of VIP ≥1, being the most relevant features (**Supplementary Figure 3**).

## Discussion

This study shows that an unbalanced peripheral blood immune gene expression is associated with hypoxemia in RSV-infected children. Children with severe hypoxemia exhibited both elevated *CXCL8* and decreased expression of antiviral genes (*ISG15*, *IFIT1*, *RIGI*, *IFNβ*, *CCL5*, and *CXCL10*), with *CXCL8*, *ISG15*, *IFIT1*, *RIGI*, and *CXCL10* being the most relevant markers. To our knowledge, no previous data exist on the association between gene expression in PBMC and hypoxemia in children infected with RSV. Our preliminary findings provide valuable insights into the pathology of RSV infection and could help develop additional therapies for a more effective antiviral response.

Our findings are particularly relevant from an immunopathological point of view, given the significant association between adequate oxygenation and the overall prognosis of RSV-infected children (3, 17). Respiratory viruses cause direct damage to cells in the airways and lungs and trigger a robust inflammatory response (17). This response can lead to swelling, fluid accumulation in the alveoli, and thickening of the airway walls, further impeding oxygen exchange (18). This issue is particularly pronounced in young children with narrower airways than adults. Additionally, infants possess weaker respiratory muscles, making it more challenging to cough and clear mucus from their airways.

Respiratory epithelial cells are the primary targets of RSV. Following infection, these cells release various cytokines, chemokines, and other immune-related genes. These molecules attract and activate immune system cells, including monocytes, neutrophils, and lymphocytes, from the bloodstream to the infection site (18). This process amplifies the immune response and initiates adaptive immunity (7). The close connection between the respiratory tract, especially the lungs, and the circulatory system supports the analysis of blood cell gene expression as a viable method for gaining insights into the immune and inflammatory response in the lungs.

Consistent with our findings, increased levels of the chemokine CXCL8 (also known as IL8) in plasma, bronchial secretions, and nasopharyngeal aspirates have been associated with the severity of RSV bronchiolitis in infants, as evidenced by the need for oxygen or mechanical ventilation, Silverman scores, and hypoxemia (19–22). CXCL8 plays a crucial role in the immune response by attracting and activating neutrophils at the infection site (23). Neutrophils are the predominant immune cells recruited to the airways in infants hospitalized with severe bronchiolitis (7, 24). Moreover, significant neutrophil infiltration in the airways has been observed through the histopathological analysis of postmortem samples from fatal cases of RSV-induced bronchiolitis (25). These findings suggest that CXCL8 may be implicated in the pathogenesis of RSV by promoting the excessive recruitment of neutrophils to the lungs (18, 24). Neutrophil extracellular traps (NETs), when released into the extracellular space of the airways, have been observed to coincide with lung injury and airway obstruction, which are associated with the worsening of respiratory diseases (24).

Conversely, the observed downregulation of genes associated with the interferon signaling pathway in the hypoxemic group, including *ISG15*, *IFIT1*, *RIGI*, and *IFNβ* itself, is consistent with a potentially compromised antiviral and immune response in these children, which may be associated with increased susceptibility to RSV infections. Specifically, RIGI is recognized as a crucial pattern recognition receptor that initiates the antiviral response against RSV (26). RIGI-mediated signaling leads to IRF3, IRF7, and NF-κB transcription factor activation and subsequent production of several cytokines and IFN types I and III. In turn, IFNs induce the production of hundreds of ISGs, including ISG15, IFIT1, and RIGI itself, in a process that aims to control virus infection (8). ISG15 and IFIT1 are classical ISGs upregulated in RSV infections (27, 28). ISG15 is associated with reduced virus production through protein ISGylation (27). IFIT1 possesses broad-spectrum antiviral functions, inhibiting viral RNA translation (29). Therefore, the antiviral response seems critical to controlling RSV infection and disease progression. Consequently, topical administration of recombinant IFN-α-2a has been found to mitigate the upper respiratory tract symptoms during RSV infection (30). Studies of nasopharyngeal samples from children with RSV infection showed that a reduction in *IFNβ* correlates with increased viral shedding (31) and a higher RSV load is associated with an increased risk of developing severe bronchiolitis (32).

The chemokines CCL5 and CXCL10, although not classified as traditional ISGs, can also be regulated by IFN (33). CCL5 (also known as RANTES) plays a pivotal role in the host immune response by attracting monocytes and T cells to the site of infection, which may contribute to RSV control (18). Consistent with our findings, other studies have also identified an inverse association of CCL5 levels in plasma (20), nasopharyngeal aspirates (34), and peripheral blood cells (35) with the severity of RSV infection. This suggests that low *CCL5* gene expression could significantly impact the immune response necessary for controlling RSV infection. This impact could be due not only to its role as a chemokine that attracts immune cells to the site of infection (18) and promotes sustained CD8^+^ T-cell responses (36) but also to its direct antiviral effect that inhibits the interaction between epithelial cells and the RSV fusion protein (37).

CXCL10 (also known as IP10) promotes antiviral response and has been linked to RSV clearance in the airways (18). In RSV-infected mice, antibody-mediated neutralization of CXCL10 resulted in increased airway hyperresponsiveness and heightened expression of mucus genes, decreased the number and maturation of dendritic cells, and reduced CD8^+^ T cells specific to RSV. These changes were related to impaired viral clearance (38). In our study, children experiencing severe hypoxemia exhibited lower levels of *CXCL10* gene expression in their peripheral blood. Accordingly, previous studies have reported an inverse association between CXCL10 levels in the nasopharynx and the severity of bronchiolitis, particularly regarding hospitalization rates in RSV-infected children (39–41).

RSV clinical scores also include signs of respiratory distress (such as respiratory rate and work of breathing), which is a major factor in determining hospitalization (along with SpO_2_) and intubation. In a previous longitudinal study within the same cohort as in the current study (35), we found that baseline *TNFα* and *CCL5* gene expressions were associated with the clinical course of RSV bronchiolitis, as evaluated with the WDS and BROSJDD scores. However, the current cross-sectional study found no significant association between the blood biomarkers and the clinical scores (data not shown). These differences between the two studies might be related to their differing designs: longitudinal and cross-sectional.

Our study identifies increased CXCL8 expression in hypoxemic infants and decreased antiviral gene expression (mainly ISGs), including *ISG15*, *IFIT1*, *RIGI*, and *IFNβ*. This observation is consistent with an unbalanced immune response in cases of severe hypoxemia, adding new insight to our findings. The exact mechanism through which the antiviral response is reduced in children with severe disease caused by RSV remains unclear. However, it is important to note that RSV can inhibit the production and signaling of IFN through its non-structural proteins (42).

### Study limitations

Our study has a series of limitations that must be considered. Firstly, the sample size is low, which could affect the study’s validity and the statistical power of the analysis. Secondly, this cross-sectional study may have introduced biases, such as the causality bias, as it does not clarify whether gene expression is a cause or effect of low oxygen. Third, no additional patient cohort was available to validate these results. Fourth, respiratory mucosa samples were not available to evaluate gene expression of the innate response. Fifth, approximately 70% of patients were of Caucasian origin, so the applicability of our data to other populations may be limited. Finally, we did not evaluate the impact of RSV treatments. However, only 4 out of 121 children received Synagis, and none received Beyfortus, so this treatment is not expected to affect the study results significantly. Additionally, two out of the four children treated had hypoxemia.

### Conclusion

Our study suggests that an imbalance favoring inflammation over antiviral defense may contribute to the pathogenesis of severe hypoxemia in RSV-infected children. Our findings highlight the complex interplay between antiviral and inflammatory responses in RSV infection, underscoring the need for a balanced immune response to combat the virus and prevent adverse outcomes effectively. Further research is needed to elucidate the specific mechanisms through which these genes influence disease severity. This could lead to identifying potential therapeutic targets to modulate the immune response in RSV infections, ultimately improving the care and outcomes of infected children.

## Data Availability

The original contributions presented in the study are included in the article/**Supplementary Materials**, further inquiries can be directed to the corresponding author/s.
