# Supplementary material for: Reduced antiviral gene expression and elevated CXCL8 expression in peripheral blood are associated with severe hypoxemia in RSV-infected children
Source: Front Immunol. 2024 Sep 30;15:1438630. doi: 10.3389/fimmu.2024.1438630 (PMC11472821; doi:10.3389/fimmu.2024.1438630)

**Supplementary Table 1**. Summary of variables included in the Wood Downes Score (WDS). WDS ranges from 1 to 10, stratifying the population into mild (≤3), moderate (4-7), and severe (≥8) bronchiolitis.

|  | **0** | **1** | **2** | **3** |
| --- | --- | --- | --- | --- |
| **Wheezing** | No | End of exhalation | During exhalation | Exhalation and inspiration |
| **Retractions** | No | Subcostal or intercostal | Supraclavicular and nasal flaring | Suprasternal and intercostal |
| **Air intake** | Normal and symmetrical | Reduced and symmetrical | Very reduced | Silent |
| **Cyanosis** | No | Present |  |  |
| **Respiratory rate** | <30 | 31-45 | 46-60 | >60 |
| **Heart rate** | <120 | >120 |  |  |

**Supplementary Table 2**. Summary of variables included in the Sant Joan de Déu Bronchiolitis score (BROSJDD). This BROSJDD score ranges from 1 to 16, stratifying the population into mild (≤5), moderate (6-10), and severe (≥11) bronchiolitis.

| **Wheezing/rales** | 0: No  1: expiratory wheezing, inspiratory rales  2: expiratory and inspiratory wheezing/rales | | | |
| --- | --- | --- | --- | --- |
| **Retraction** | 0: No  1: Subcostal, Lower Intercostal  2: Subcostal, Lower Intercostal + supraclavicular + nasal flaring  3: Subcostal, Lower Intercostal + supraclavicular + nasal flaring + Upper Intercostal + Suprasternal | | | |
| **Air intake** | 0: Normal  1: Reduced and symmetrical  2: Asymmetrical  3: Very reduced | | | |
| **Oxygen saturation** | Without supplementary oxygen:  0: >95%  1: 91-94%  2: <90% | | With supplementary oxygen:  1: >94% with FiO2<40%  2:<94% with FiO2>40% | |
| **Respiratory rate** | 0  <3 m: <40  3-12m:<30  12-24m: <30 | 1  40-60  30-50  30-40 | 2  60-70  50-60  40-50 | 3  >70  >60  >50 |
| **Heart rate** | 0  <1 y: <130  1-2 y:<110 | 1  130-150  110-120 | 2  150-170  120-140 | 3  >170  >140 |

**Abbreviation**: FiO2, inspired fraction of oxygen: m, months old; y, years old.

**Supplementary Figure 1**. Normalization of gene expression of *IL6, TNFα, CXCL8, ISG15, IFIT1, RIG-I, IFNβ, CCL5*, and *CXCL10* in peripheral blood in RSV-infected children. **Statistics**: Gene expression values were normalized using log-transformation (log_10_) and scaling by mean centering using MetaboAnalyst 6.0 (<https://www.metaboanalyst.ca/>). **Abbreviations**: RSV, respiratory syncytial virus; IL6, Interleukin 6; TNFα, Tumor necrosis factor-alpha; CXCL8, Chemokine C-X-C motif ligand 8; ISG15, Interferon-stimulated gene 15; IFIT1, Interferon-induced protein with tetratricopeptide repeats 1; RIGI, Retinoic acid-inducible gene I; IFNβ, Interferon-β1; CCL5, Chemokine C-C motif ligand 5; CXCL10, Chemokine C-X-C motif ligand 10.


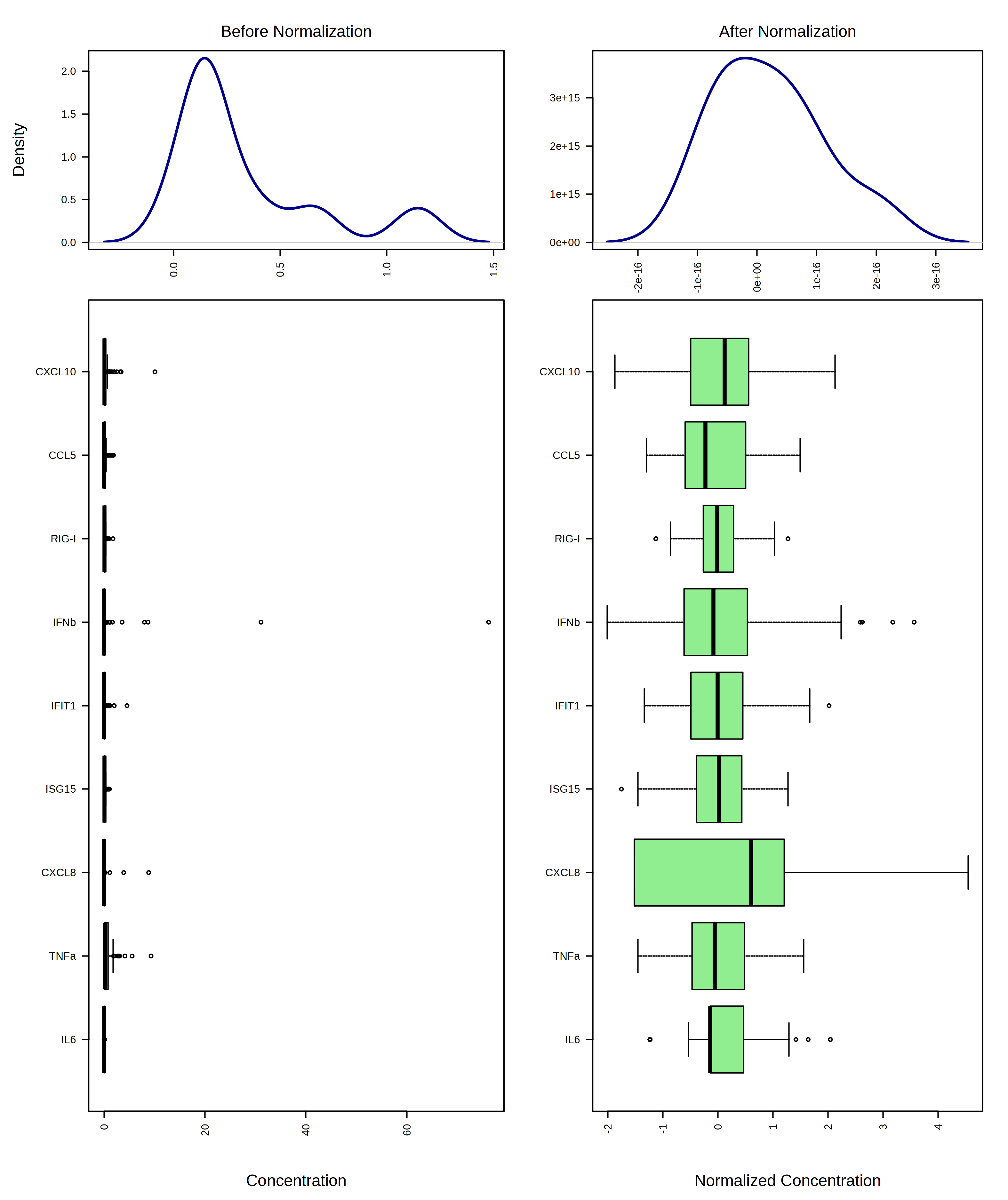


**Supplementary Figure 2.** Permutation plot for validation of the OPLS-DA model. **Statistics**: A permutation test was used to confirm the validity of the OPLS-DA model, with a permutation number of 1000, using MetaboAnalyst 6.0 (<https://www.metaboanalyst.ca/>). R2Y represents the model interpretation rate; Q2 indicates the predictive ability of the model; R2Y and Q2, closer to 1, indicate that the model is more stable and reliable. **Abbreviations**: OPLS-DA, orthogonal partial least squares discriminant analysis; p-value, level of significance.


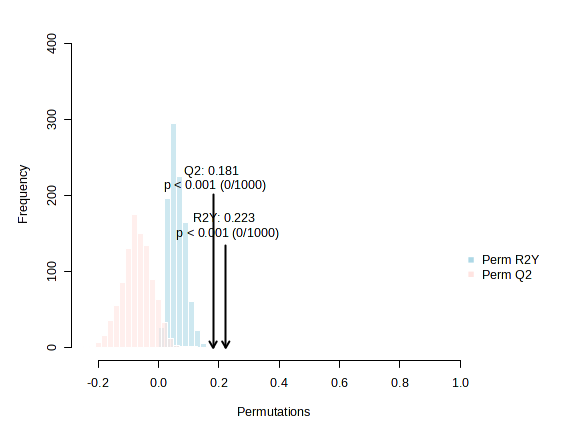


**Supplementary Figure 3**. VIP values summary of gene expression in peripheral blood for severe hypoxemia (SpO_2_ ≤90%) in RSV-infected children. **Statistics**: Data were calculated using an OPL-DA. **Abbreviations**: VIP, Variable importance in projection; RSV, respiratory syncytial virus; SpO_2_; saturation of peripheral oxygen; OPLS-DA, orthogonal partial least squares discriminant analysis; IL6, Interleukin 6; TNFα, Tumor necrosis factor-alpha; CXCL8, Chemokine C-X-C motif ligand 8; ISG15, Interferon-stimulated gene 15; IFIT1, Interferon-induced protein with tetratricopeptide repeats 1; RIGI, Retinoic acid-inducible gene I; IFNβ, Interferon-β1; CCL5, Chemokine C-C motif ligand 5; CXCL10, Chemokine C-X-C motif ligand 10.


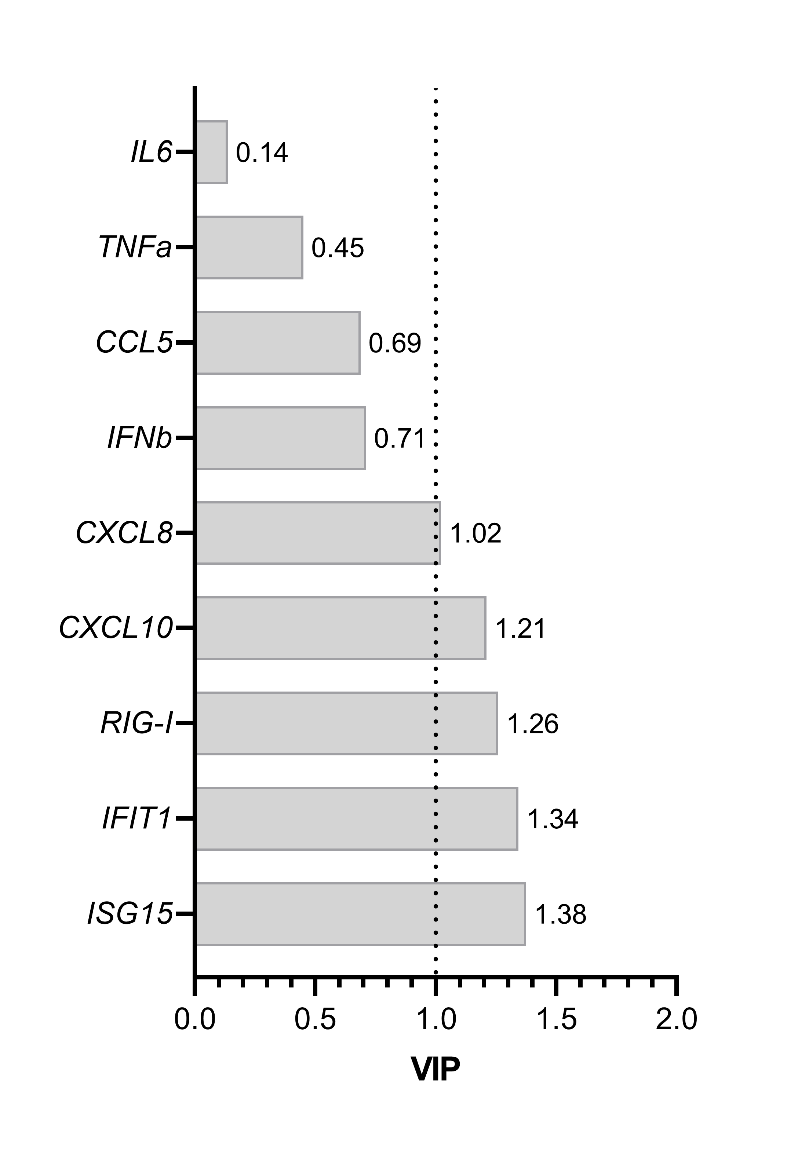

Supplement: Supplementary file 1 [file DataSheet1.docx]
